# Serum and urinary biomarkers in lupus nephritis: do suPAR and VEGF play a role?

**DOI:** 10.1590/2175-8239-JBN-2024-0137en

**Published:** 2025-06-13

**Authors:** Tamires Teixeira Piraciaba, Michelle Tiveron Passos Riguetti, Gianna Mastroianni Kirsztajn

**Affiliations:** 1 Universidade Federal de São Paulo, Departamento de Medicina, São Paulo, SP, Brazil.

**Keywords:** Vascular Endothelial Growth Factor, suPAR, Lupus Erythematosus, Systemic, Lupus Nephritis, Hematuria, Proteinuria, Biomarkers

## Abstract

**Introduction:**

The extent, activity, and effects of renal involvement in systemic lupus erythematosus are conditions difficult to diagnose and a renal biopsy is usually required for such purpose. Non-invasive methods are then of high interest to better understand lupus nephritis (LN) features and prognosis. We have evaluated VEGF and suPAR levels as non-invasive biomarkers of disease activity in LN.

**Methods:**

This cross-sectional study enrolled patients with LN aged 18 years or older of both genders who were assisted at the Outpatient Clinic of Glomerular Diseases of the Division of Nephrology of the Federal University of São Paulo (UNIFESP). Clinical renal histopathological characteristics, routine laboratory test profiles, and serum and urinary suPAR and serum VEGF of patients with LN were evaluated.

**Results:**

The sample consisted of 51 patients with LN, most of them females (88.2%), white (51.0%) with a mean age of 36.1 years (18 to 65 years), and a median disease duration of 2.5 years (1 month to 32 years). A positive correlation of urinary suPAR with SLEDAI-2K scores and hematuria was found. No significant correlation was found between serum VEGF and serum and urinary suPAR and proteinuria, serum creatinine, and urinary protein/creatinine ratio.

**Conclusions:**

Serum suPAR and VEGF levels were not indicative of renal or general SLE activity, but our data suggest that urinary suPAR may be a useful biomarker of such conditions, as its levels have been shown to correlate with the presence of hematuria and with higher SLEDAI-2K scores.

## Introduction

The extent, activity, and impact of renal involvement in systemic lupus erythematosus (SLE) are difficult to diagnose. No single clinical parameter or laboratory test defines such features, although some of them contribute to an adequate diagnosis. Given the complexity of the disease, an objective assessment using scores, such as the Systemic Lupus Erythematosus Disease Activity Index (SLEDAI), is useful to detect clinical changes, analyze new possible therapies, and guide the control of disease activity^1^.

Renal involvement occurs in about 60% of SLE patients^2^ and kidney biopsy is the gold standard for diagnosis of lupus nephritis (LN). Because biopsy is an invasive procedure, noninvasive methods are of great interest to better establish LN features, including flares and prognosis^3^. An ideal biomarker for LN should reflect disease activity and histological classes, predict relapses, be easily measured, and enable early diagnosis of renal involvement^4^.

Among the biomarkers that were recently suggested as laboratory tools in the management of LN are vascular endothelial growth factor (VEGF) and soluble urokinase activating plasminogen receptor (suPAR).

VEGF is a biomarker produced by monocyte endothelial cells, macrophages, activated T cells, among other cells. Its production is increased as a response to inflammation, but its renal expression is reduced in patients with LN. Therefore, the role of VEGF is controversial and still needs to be elucidated^5^.

Many cytokine studies in SLE have focused on a limited number of markers. Adhya et al.^6^ assessed a panel of six cytokines and concluded that tumor necrosis factor receptor 1 (TNF-R1) especially in serum but also in urine could be a useful marker along with serum VEGF and monocyte chemoattractant protein 1 (MCP-1), particularly for diagnosing active LN.

Urokinase activating plasminogen receptor (uPAR) is a membrane-bound protein present in several cells involved in the immune system (macrophages, monocytes, T lymphocytes)^7^. When dissociated from the cell surface, its soluble form (suPAR) is created. In podocytes, uPAR promotes cell contraction, fusion of podocyte processes, and proteinuria^8^, and has been described as an indicator of an activated immune system. This biomarker has been studied in different diseases^9^ and its role needs to be better evaluated in LN.

Enocsson et al.^10^ first demonstrated an association between serum suPAR levels and irreversible organ damage in SLE. Significantly higher suPAR levels were observed in patients with a higher Systemic Lupus International Collaborating Clinics (SLICC)/American College of Rheumatology (ACR) Damage Index (SDI) classification 2–5 years after their inclusion.

In the present study, we evaluated VEGF and suPAR levels as candidates for non-invasive biomarkers of disease activity in LN and assessed their association with traditional laboratory tests used to monitor disease activity in SLE and LN.

## Methods

### Study Design, Working Definition, Clinical and Laboratory Assessments

This prospective cross-sectional study enrolled adult patients with LN aged 18 years or older, of both genders, assisted at the Outpatient Clinic of Glomerular Diseases of the Division of Nephrology of the Federal University of São Paulo (UNIFESP).

Clinical, laboratory, and renal histopathological characteristics of the patients were evaluated, as well as routine laboratory tests used for patient follow-up. The study endpoints were serum VEGF and serum and urinary suPAR as markers of disease activity in individuals with LN. Both biomarkers were studied in the same urine and blood samples in which the routine tests were performed. SLEDAI-2K criteria were used for establishing LN activity scores.

SLE diagnosis was established according to the American Association of Rheumatology criteria^11^. Acute kidney injury (AKI) was defined according to serum creatinine elevation, i.e., increase of 1.5 to 1.9 times the baseline value over seven days or absolute increase of 0.3 mg/dL within 48 h, as proposed by KDIGO^12^.

### Determination of VEGF

VEGF levels were determined using the Quantikine^®^ ELISA Human VEGF kit (cat. DVE00) according to the manufacturer’s instructions.

On the plate containing the solid phase and the VEGF monoclonal antibody, 100 µL of RD1W diluent, the standard curve, the controls (Immunoassay Control Group 1, R&D Systems, cat. # QC01), and the samples per well were incubated for 2 h at room temperature.

Washings were performed and 200 µL of Human VEGF conjugate was added and incubated for 2 h at room temperature. The plate was then washed and 200 µL of the substrate solution was added and incubated for 25 min at room temperature. After the incubation period, the reaction was blocked by the addition of 50 µL of the stop solution.

The reaction reading was performed on a spectro­photometer (Titertek Multiskan, UNISCIENCE) at the 450 nm wavelength.

### Determination of suPAR

The determination of suPAR levels was performed using a commercial kit according to the manufacturer’s instructions.

To the plate containing the solid phase adsorbed with uPAR monoclonal antibody, 100 µL of the RD1W diluent, 50 µL of the standard, the controls (Immunoassay Control Group 4, R & D Systems, cat. # QC21), and the samples were added, and the plate was incubated for 2 h at room temperature. Washings were performed and 200 µL of Human uPAR Conjugate was added and incubated for 2 h at room temperature. The plate was then washed and 200 µL of the substrate solution was added and incubated for 30 min at room temperature. After the incubation period, the reaction was blocked by the addition of 50 µL of the stop solution. The reaction reading was performed on a spectrophotometer (Titertek Multiskan, UNISCIENCE) at 450 nm wavelength.

### Statistical analysis

A descriptive statistical analysis was performed first, followed by inferential analyses, using Mann-Whitney (urinary and serum suPAR and serum VEGF vs. hematuria, anti-DNA and complement levels) and Kruskal-Wallis tests (urinary and serum suPAR and serum VEGF vs. proteinuria levels), and Spearman correlation (urinary and serum suPAR and serum VEGF according to the SLEDAI-2K scores). The significance level was established as p < 0.05. Statistical analyses were performed using the statistics program R version 3.3.2.

## Results

The sample consisted of 51 patients with LN, most of them females (88.2%), white (51.0%), with a median age of 32 years, ranging from 18 to 65 years, and a median disease duration of 2.5 years, ranging from 1 month to 32 years (Table 1).

**Table 1:** General characteristics of the patients with lupus nephritis

Gender (n = 51)	female	45	88.2%
	male	6	11.8%
Race/ethnicity (n = 51)	white	26	51.0%
	black	10	19.6%
	mixed (black+white)	15	29.4%
Age (years) (n = 51)	mean	36.1
	median	32.0
	minimum-maximum	18–65
	standard deviation	12.2
LN classes (n = 51)	III	7	13.7%
	IV	27	53.0%
	V	16	31.4%
	unknown	1	2.0%
Time of disease (years) (n = 51)	mean	5.72
	median	2.50
	minimum-maximum	0.08–32.00
	standard deviation	7.46
Immuno­suppressive drugs (n = 51)	CSA	1	2.0%
	PRED	17	33.3%
	AZA+PRED	5	9.8%
	MYC+PRED	25	49.0%
	None	3	5.9%
Acute kidney injury (n = 51)	no	44	86.3%
	yes	7	13.7%
SLEDAI-2K score (n = 51)	mean	10.2
	median	11.0
	minimum-maximum	0.0–23.0
	standard deviation	5.6

Abbreviation – LN: lupus nephritis; AZA: azathioprine; CSA: cyclosporine; MYC: mycophenolate; PRED: prednisone. Note – Most patients had LN classes IV (51.0%) and V (31.4%), predominantly without acute kidney injury (86.3%).

Routine laboratory results are shown in Table 2, and urinary suPAR, serum suPAR, and serum VEGF levels in Table 3.

**Table 2:** Laboratory results of the patients with lupus nephritis

Hematuria (n = 51)	yes	28	54.9%
	no	23	45.1%
Proteinuria (g/24h) (n = 51)	< 1	23	45.1%
	1–3.5	11	21.6%
	> 3.5	17	33.3%
Proteinuria (g/24h) (n = 51)	mean	2.50
	median	1.05
	minimum-maximum	0.00–9.49
	standard deviation	2.64
Anti-DNA (n = 51)	positive	17	33.3%
	negative	34	66.7%
Complement (n = 51)	normal	35	68.6%
	low	16	31.4%
Serum albumin (g/dL) (n = 51)	normal	34	66.7%
	low	17	33.3%
Serum creatinine (mg/dL) (n = 51)	< 1.2 (normal)	36	70.6%
	≥ 1.2	15	29.4%
Serum creatinine (mg/dL) (n = 51)	mean	1.26
	median	0.97
	minimum-maximum	0.43–4.88
	standard deviation	0.98
uPCR (g/g) (n = 51)	< 0.15 (normal)	7	13.7%
	≥ 0.15	44	86.3%
uPCR (g/g) (n = 51)	mean	2.05
	median	1.04
	minimum-maximum	0.00–9.69
	standard deviation	2.46

Abbreviation – uPCR: urinary protein/creatinine ratio.

**Table 3:** Urinary and serum supar and serum vegf levels of patients with lupus nephritis

Urinary suPAR (pg/mL) (n = 51)	mean	3445.01
	median	2687.70
	minimum-maximum	453.40–12440.23
	standard deviation	2612.49
Serum VEGF (pg/mL) (n = 51)	mean	303.97
	median	225.37
	minimum-maximum	2.26–1261.13
	standard deviation	297.13
Serum suPAR (pg/mL) (n = 51)	mean	3525.23
	median	3098.19
	minimum-maximum	1326.30–14591.21
	standard deviation	2137.21

It was found that 28 (54.9%) patients had glomerular hematuria characterized by the presence of a dysmorphic pattern of red cells in urine. Proteinuria levels above 3.5 g/24 h were observed in 17 (33.3%) patients.

Negative anti-DNA and normal serum albumin were confirmed in 34 (66.7%) patients. Of the total, 16 (31.4%) patients exhibited complement consumption (serum levels of C3 and/or C4 below the normal reference range).

The association of the three biomarkers (urinary suPAR, VEGF, and serum suPAR) with hematuria, 24-hour proteinuria, positivity for anti-DNA antibodies, serum complement levels, serum creatinine, urine protein/creatinine ratio, and SLEDAI-2K scores was evaluated. Inferential results confirmed that patients with glomerular hematuria had higher urinary suPAR levels compared to those without hematuria (p = 0.003). For VEGF (p = 0.219) and serum suPAR (p = 0.623), there was a negative association that was not statistically significant.

There was no association between proteinuria levels and urinary suPAR (p = 0.631), VEGF (p = 0.727), and serum suPAR (p = 0.561). Although patients with positive anti-DNA showed lower urinary suPAR (p = 0.936), VEGF (p = 0.424), and serum suPAR (p = 0.328) compared to those with negative anti-DNA, this was not statistically significant. Categories of serum creatinine and the protein/creatinine ratio were not associated with suPAR and VEGF levels.

**Figure 1.**
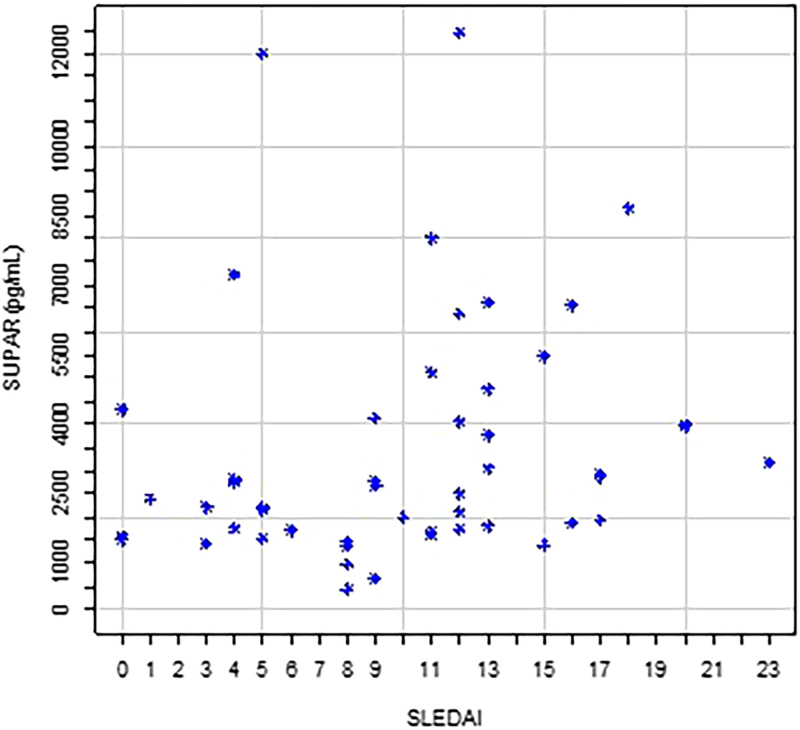
Bidimensional dispersion diagram between urinary suPAR (pg/mL) and SLEDAI-2K scores in patients with lupus nephritis.

We confirmed a positive correlation between SLEDAI-2K and urinary suPAR (Spearman correlation coefficient r = 0.347; p = 0.012, Figure 1), and also between glomerular hematuria and urinary suPAR (r = 0.322; p = 0.021). The higher the SLEDAI-2K and hematuria counts, the higher the urinary suPAR levels.

No significant association was found between serum VEGF and urinary suPAR (r = −0.197; p = 0.165), proteinuria (r = −0.187; p = 0.188), serum creatinine (r = 0.034; p = 0.814), and urinary protein/creatinine ratio (r = −0.220; p = 0.121).

The same occurred when investigating potential associations between serum suPAR and SLEDAI-2K (r = 0.055; p = 0.699), 24-hour proteinuria levels (r = 0.154; p = 0.280), and the protein/creatinine ratio (r = 0.253; p = 0.073). Higher levels of serum creatinine were associated with greater levels of serum suPAR (r = 0.511; p < 0.001).

## DISCUSSION

Lupus nephritis is one of the most severe manifestations of SLE, leading to a high disease morbidity and mortality. The assessment of kidney injury activity and severity is important for a better follow-up of the patients. This study aimed to evaluate the profile of suPAR and VEGF in patients with LN and their eventual association with disease activity in SLE and LN.

The sample of the LN population here evaluated consisted of 51 patients, 88.2% female, with an average age of 36.1 years. These characteristics are similar to those of other studies. Satirapoj et al.^13^ studied 68 Asian patients with a predominance of females (97.1%) and with a mean age of 31.7 years. In 2017, a Brazilian study analyzed 8,761 causes of death in which SLE was recorded as the underlying disease. In that study, 90.7% of the sample were women, 49.2% were white, and the age range was 20 to 39 years^14^.

The race/ethnicity of the subjects is difficult to classify in Brazil due to the high miscegenation of the population. Nevertheless, based on the assigned race of the patients in the medical records, 51% were white, a similar percentage to that of the last study cited^14^.

In our service, LN patients are usually submitted to kidney biopsy when they present with more prominent urinary abnormalities and/or renal dysfunction at presentation and/or do not respond to immunosuppressive treatment during follow-up. Regarding the histological diagnosis, there was a predominance of patients with class IV LN (51%), followed by class V LN (31.4%). Class IV is the histological pattern associated with more severe LN, as reported by other studies^13, 15, 16^. It is worth noting that our study was performed in a tertiary care hospital that receives patients from all over the country, often referred for severe and complex conditions, which in part justifies the predominance of such a presentation. Although LN class IV was the predominant histological class, only a minority of these patients (13.7%) had AKI at some point in the course of the disease.

Women with class IV and moderate activity predominated in the study population (according to SLEDAI-2K). SLEDAI-2K is a modification of the original SLEDAI, and both are indicators of mortality in SLE and are used to measure disease activity and assess the need for treatment initiation^17^.

In this study, a positive and increasing correlation was found between SLEDAI-2K scores and urinary suPAR levels (the higher the score, the higher the urinary biomarker values), but not with serum suPAR and VEGF levels.

In our population, the anti-DNA antibody test was positive in 33.3% and hypocomplementemia (decreased C3 or C4) occurred in 31.4% of the patients. Complement levels and anti-DNA reactivity were not correlated with suPAR or VEGF levels. It is known that positive or increasing anti-DNA antibody titers and hypocomplementemia, especially low C3 levels, are indicative of renal activity, but these findings are neither essential nor sufficient for such diagnosis^18^.

Glomerular hematuria alone is an important urinary abnormality in the diagnosis and follow-up of patients with LN and in this study, hematuria was present in 54.9% of the patients by the time of the samples collection. Although often associated with renal activity, hematuria does not always predict active disease in LN^19^, and additional tests are needed to establish renal involvement due to LN. Concurrent elevated urinary suPAR and hematuria would reinforce renal activity secondary to SLE.

In the evaluation of 87 subjects with LN, a significant correlation was found between active LN and urinary VEGF, but not serum VEGF levels^6^.

The biomarkers here evaluated have also been studied in other glomerulopathies, such as focal segmental glomerulosclerosis. Li et al.^20^ evaluated serum suPAR in 109 individuals and observed that its levels were significantly higher in patients with such disease than in healthy subjects or in patients with minimal change disease or with membranous nephropathy.

In the present study, we evaluated traditional and new laboratory biomarkers in patients with LN. There was no association between serum suPAR and VEGF levels and renal or general SLE activity, but urinary levels of suPAR correlated with the presence of glomerular hematuria and with higher SLEDAI-2K scores, i.e. with LN and SLE activity.

Limitations of this study include the peculiarities of the Brazilian ethnicity that could be related to polymorphisms of the VEGF gene and susceptibility to SLE. We did not evaluate subgroups of patients according to time of disease, types of treatment, biopsy findings, and other parameters, because a higher number of patients would be necessary to compose representative subgroups. Besides, although patients were recruited early in the course of the disease, with a mean of 2.5 years from diagnosis to inclusion in the study, different lengths of time on antimalarials and/or immunosuppressants may have an impact on the results.

There are few studies investigating the role of suPAR and VEGF in glomerular diseases, particularly in LN. These initial results suggest a potential role for urinary suPAR as a biomarker that could reflect LN and SLE activity, which could be used as an additional tool in clinical practice and is available for laboratory determination in an easily collected urine sample.

## Data Availability

Data that are not presented here and corroborate the results of this study will be available upon request to the corresponding author.
