# Supplementary material for: Serum and urinary biomarkers in lupus nephritis: do suPAR and VEGF play a role?
Source: J Bras Nefrol. 2025 Jun 13;47(4):e20240137. doi: 10.1590/2175-8239-JBN-2024-0137en (PMC12165717; doi:10.1590/2175-8239-JBN-2024-0137en)
Supplement: Translation

# Translation: Biomarcadores séricos e urinários na nefrite lúpica: qual o papel do suPAR e do VEGF?

**DOI:** 10.1590/2175-8239-JBN-2024-0137pt

**Authors:** Tamires Teixeira Piraciaba, Michelle Tiveron Passos Riguetti, Gianna Mastroianni Kirsztajn

**Affiliations:** 1 Universidade Federal de São Paulo, Departamento de Medicina, São Paulo, SP, Brasil.

**Keywords:** Fator de Crescimento do Endotélio Vascular, suPAR, Lúpus Eritematoso Sistêmico, Nefrite Lúpica, Hematúria, Proteinúria, Biomarcadores

## Abstract

**Introdução:**

A extensão, atividade e efeitos do acometimento renal no lúpus eritematoso sistêmico são condições de difícil diagnóstico, sendo geralmente necessária biópsia renal para tal fim. Métodos não invasivos despertam, portanto, grande interesse para uma melhor compreensão das características e prognóstico da nefrite lúpica (NL). Avaliamos os níveis de VEGF e suPAR como biomarcadores não invasivos da atividade da doença na NL.

**Métodos:**

Este estudo transversal envolveu pacientes com NL, de ambos os sexos, com 18 anos ou mais, atendidos no Ambulatório de Glomerulopatias da Disciplina de Nefrologia da Universidade Federal de São Paulo (UNIFESP). Foram avaliadas características clínicas e histopatológicas renais, perfis de exames laboratoriais de rotina, suPAR sérico e urinário e VEGF sérico de pacientes com NL.

**Resultados:**

A amostra consistiu em 51 pacientes com NL, a maioria do sexo feminino (88,2%), brancos (51,0%) com idade média de 36,1 anos (18 a 65 anos) e duração mediana da doença de 2,5 anos (1 mês a 32 anos). Foi encontrada uma correlação positiva entre suPAR urinário e escores SLEDAI-2K e hematúria. Não foi observada correlação significativa entre VEGF sérico e suPAR sérico e urinário e proteinúria, creatinina sérica e relação P/C urinária.

**Conclusões:**

Os níveis séricos de suPAR e VEGF não foram indicativos de atividade renal ou geral do LES, mas nossos dados sugerem que o suPAR urinário pode ser um biomarcador útil para tais condições, uma vez que seus níveis demonstraram correlacionar-se com a presença de hematúria e com escores SLEDAI-2K mais elevados.

## Introdução

A extensão, a atividade e o impacto do acometi­mento renal no lúpus eritematoso sistêmico (LES) são difíceis de diagnosticar. Nenhum parâmetro clínico ou exame laboratorial isolado define essas características, embora alguns deles contribuam para um diagnóstico adequado. Dada a complexidade da doença, uma avaliação objetiva utilizando escores, como o Índice de Atividade da Doença do Lúpus Eritematoso Sistêmico (SLEDAI, na sigla em inglês), é útil para detectar alterações clínicas, analisar novas possíveis terapias e orientar o controle da atividade da doença^1^.

O acometimento renal ocorre em cerca de 60% dos pacientes com LES^2^ e a biópsia renal é o padrão-ouro para o diagnóstico de nefrite lúpica (NL). Como a biópsia é um procedimento invasivo, métodos não invasivos são de grande interesse para melhor estabelecer as características da NL, incluindo surtos e prognóstico^3^. Um biomarcador ideal para a NL deve refletir a atividade da doença e as classes histológicas, prever recidivas, ser facilmente mensurável e permitir o diagnóstico precoce do acometimento renal^4^.

Entre os biomarcadores recentemente sugeridos como ferramentas laboratoriais no manejo da NL estão o fator de crescimento endotelial vascular (VEGF) e o receptor do ativador de plasminogênio tipo uroquinase solúvel (suPAR).

O VEGF é um biomarcador produzido por células endoteliais, monócitos, macrófagos, células T ativadas, entre outras células. Sua produção é aumentada em resposta à inflamação, mas sua expressão renal é reduzida em pacientes com NL. Portanto, o papel do VEGF é controverso e ainda precisa ser elucidado^5^.

Muitos estudos sobre citocinas no LES têm-se concentrado em um número limitado de marcadores. Adhya et al.^6^ avaliaram um painel de seis citocinas e concluíram que o receptor 1 do fator de necrose tumoral (TNF-R1), especialmente no soro, mas também na urina, pode ser um marcador útil, juntamente com o VEGF sérico e a proteína quimiotática de monócitos-1 (MCP-1), particularmente para o diagnóstico de NL ativa.

O receptor do ativador de plasminogênio tipo uroquinase (uPAR) é uma proteína ligada à membrana presente em diversas células envolvidas no sistema imunológico (macrófagos, monócitos, linfócitos T)^7^. Quando dissociada da superfície celular, dá origem à sua forma solúvel (suPAR). Nos podócitos, o uPAR promove a contração celular, a fusão dos processos podocitários e a proteinúria^8^, e foi descrito como um indicador de ativação do sistema imunológico. Este biomarcador tem sido estudado em diferentes doenças^9^ e seu papel precisa ser melhor avaliado na NL.

Enocsson et al.^10^ foram os primeiros a demonstrar uma associação entre os níveis séricos de suPAR e danos irreversíveis aos órgãos no LES. Níveis significativamente mais elevados de suPAR foram observados em pacientes com uma classificação mais elevada no Índice de Danos (SDI) do Systemic Lupus International Collaborating Clinics (SLICC)/American College of Rheumatology (ACR) entre 2 e 5 anos após sua inclusão.

No presente estudo, avaliamos os níveis de VEGF e suPAR como candidatos a biomarcadores não invasivos da atividade da doença na NL e avaliamos sua associação com testes laboratoriais tradicionais utilizados para monitorar a atividade da doença no LES e na NL.

## Métodos

### Desenho do Estudo, Definição do Trabalho, Avaliações Clínicas e Laboratoriais

Este estudo transversal prospectivo incluiu pacientes adultos com NL com idade igual ou superior a 18 anos, de ambos os sexos, atendidos no Ambulatório de Glomerulopatias da Disciplina de Nefrologia da Universidade Federal de São Paulo (UNIFESP).

Foram avaliadas as características clínicas, labo­ratoriais e histopatológicas renais dos pacientes, bem como os exames laboratoriais de rotina utilizados no acompanhamento clínico. Os desfechos do estudo foram os níveis séricos de VEGF e os níveis séricos e urinários de suPAR como marcadores da ativi­dade da doença em indivíduos com NL. Ambos os biomarcadores foram analisados nas mesmas amostras de urina e sangue em que foram realizados os testes de rotina. Os critérios do SLEDAI-2K foram empregados para estabelecer os escores de atividade da NL.

O diagnóstico de LES foi estabelecido de acordo com os critérios da American Association of Rheumatology^11^. A injúria renal aguda (IRA) foi definida com base na elevação da creatinina sérica, ou seja, aumento de 1,5 a 1,9 vezes o valor basal em sete dias ou aumento absoluto de 0,3 mg/dL em 48 horas, conforme proposto pelo KDIGO^12^.

### Determinação do VEGF

Os níveis de VEGF foram determinados utilizando o kit Quantikine^®^ ELISA Human VEGF (cat. DVE00) conforme as instruções do fabricante.

Na placa contendo a fase sólida e o anticorpo monoclonal VEGF, foram incubados, por 2 horas em temperatura ambiente, 100 µL de diluente RD1W, a curva padrão, os controles (Immunoassay Control Group 1, R&D Systems, cat. # QC01) e as amostras por poço.

Foram realizadas lavagens e 200 µL de conjugado de VEGF humano foram adicionados e incubados por 2 h em temperatura ambiente. A placa foi então lavada e 200 µL da solução de substrato foram adicionados e incubados por 25 minutos em temperatura ambiente. Após o período de incubação, a reação foi bloqueada pela adição de 50 µL de solução de parada.

A leitura da reação foi realizada em um espec­trofotômetro (Titertek Multiskan, UNISCIENCE) no comprimento de onda de 450 nm.

### Determinação do suPAR

A determinação dos níveis de suPAR foi realizada usando um kit comercial de acordo com as instruções do fabricante.

Na placa contendo a fase sólida adsorvida com o anticorpo monoclonal anti-uPAR, foram adicionados 100 µL do diluente RD1W, 50 µL do padrão, os controles (Immunoassay Control Group 4, R & D Systems, cat. # QC21) e as amostras. A placa foi incubada por 2 h em temperatura ambiente. Foram realizadas lavagens e, em seguida, 200 µL de conjugado de uPAR humano foram adicionados e incubados por 2 h em temperatura ambiente. A placa foi então lavada e 200 µL da solução de substrato foram adicionados e incubados por 30 minutos em temperatura ambiente. Após o período de incubação, a reação foi bloqueada pela adição de 50 µL de solução de parada. A leitura da reação foi realizada em um espectrofotômetro (Titertek Multiskan, UNISCIENCE) no comprimento de onda de 450 nm.

### Análise Estatística

Primeiramente, foi realizada uma análise estatística descritiva, seguida de análises inferenciais, utilizando os testes de Mann-Whitney (suPAR urinário e sérico e VEGF sérico vs. níveis de hematúria, anti-DNA e de complemento) e Kruskal-Wallis (suPAR urinário e sérico e VEGF sérico vs. níveis de proteinúria), além da correlação de Spearman (suPAR urinário e sérico e VEGF sérico de acordo com os escores SLEDAI-2K). O nível de significância foi estabelecido como p < 0,05. As análises estatísticas foram realizadas utilizando o programa estatístico R versão 3.3.2.

## Resultados

A amostra foi composta por 51 pacientes com NL, a maioria do sexo feminino (88,2%), brancos (51,0%), com idade mediana de 32 anos, variando de 18 a 65 anos, e duração mediana da doença de 2,5 anos, com variação de 1 mês a 32 anos (Tabela 1).

**Tabela 1:** Características gerais dos pacientes com nefrite lúpica

Sexo (n = 51)	feminino	45	88,2%
	masculino	6	11,8%
Raça/etnia (n = 51)	brancos	26	51,0%
	pretos	10	19,6%
	miscigenados (pretos+brancos)	15	29,4%
Idade (anos) (n = 51)	média	36,1
	mediana	32,0
	mínimo-máximo	18–65
	desvio-padrão	12,2
Classes de NL (n = 51)	III	7	13,7%
	IV	27	53,0%
	V	16	31,4%
	desconhecida	1	2,0%
Duração da doença (anos) (n = 51)	média	5,72
	mediana	2,50
	mínimo-máximo	0,08–32,00
	desvio-padrão	7,46
Medicamentos imunos­supressores (n = 51)	CSA	1	2,0%
	PRED	17	33,3%
	AZA+PRED	5	9,8%
	MYC+PRED	25	49,0%
	Nenhum	3	5,9%
Injúria renal aguda (n = 51)	não	44	86,3%
	sim	7	13,7%
Score SLEDAI-2K (n = 51)	média	10,2
	mediana	11,0
	mínimo-máximo	0,0–23,0
	desvio-padrão	5,6

Abreviações – NL: nefrite lúpica; AZA: azatioprina; CSA: ciclosporina; MYC: micofenolato; PRED: prednisona.

Nota – A maioria dos pacientes apresentou NL classes IV (51,0%) e V (31,4%), predominantemente sem injúria renal aguda (86,3%).

Os resultados laboratoriais de rotina são apresentados na Tabela 2, e os níveis de suPAR urinário, suPAR sérico e VEGF sérico, na Tabela 3.

**Tabela 2:** Resultados laboratoriais dos pacientes com nefrite lúpica

Hematúria (n = 51)	sim	28	54,9%
	não	23	45,1%
Proteinúria (g/24h) (n = 51)	< 1	23	45,1%
	1–3,5	11	21,6%
	> 3,5	17	33,3%
Proteinúria (g/24h) (n = 51)	média	2,50
	mediana	1,05
	mínimo-máximo	0,00–9,49
	desvio-padrão	2,64
Anti-DNA (n = 51)	positivo	17	33,3%
	negativo	34	66,7%
Complemento (n = 51)	normal	35	68,6%
	baixo	16	31,4%
Albumina sérica (g/dL) (n = 51)	normal	34	66,7%
	baixa	17	33,3%
Creatinina sérica (mg/dL) (n = 51)	< 1,2 (normal)	36	70,6%
	≥ 1,2	15	29,4%
Creatinina sérica (mg/dL) (n = 51)	média	1,26
	mediana	0,97
	mínimo-máximo	0,43–4,88
	desvio-padrão	0,98
RPCU (g/g) (n = 51)	< 0,15 (normal)	7	13,7%
	≥ 0,15	44	86,3%
RPCU (g/g) (n = 51)	média	2,05
	mediana	1,04
	mínimo-máximo	0,00–9,69
	desvio-padrão	2,46

Abreviação – RPCU: relação proteína/creatinina urinária.

**Tabela 3:** Níveis de supar urinário e sérico e vegf sérico de pacientes com nefrite lúpica

suPAR urinário (pg/mL) (n = 51)	média	3445,01
	mediana	2687,70
	mínimo-máximo	453,40–12440,23
	desvio-padrão	2612,49
VEGF sérico (pg/mL) (n = 51)	média	303,97
	mediana	225,37
	mínimo-máximo	2,26–1261,13
	desvio-padrão	297,13
suPAR sérico (pg/mL) (n = 51)	média	3525,23
	mediana	3098,19
	mínimo-máximo	1326,30–14591,21
	desvio-padrão	2137,21

Verificou-se que 28 (54,9%) pacientes apres­entaram hematúria glomerular caracterizada pela presença de dismorfismo eritrocitário. Níveis de proteinúria superiores a 3,5 g/24 h foram observados em 17 (33,3%) pacientes.

O anti-DNA negativo e níveis normais de albumina sérica foram confirmados em 34 (66,7%) pacientes. Do total, 16 (31,4%) pacientes apresentaram consumo de complemento (níveis séricos de C3 e/ou C4 abaixo da faixa de referência de normalidade).

Avaliou-se a associação dos três biomarcadores (suPAR urinário, VEGF e suPAR sérico) com hematúria, proteinúria de 24 horas, positividade para anticorpos anti-DNA, níveis séricos de complemento, creatinina sérica, relação proteína/creatinina urinária e escores SLEDAI-2K. Os resultados inferenciais confirmaram que pacientes com hematúria glomerular apresentaram níveis mais elevados de suPAR urinário em comparação com aqueles sem hematúria (p = 0,003). Já para VEGF (p = 0,219) e suPAR sérico (p = 0,623), houve uma associação negativa, sem significância estatística.

Não houve associação entre níveis de proteinúria e suPAR urinário (p = 0,631), VEGF (p = 0,727) e suPAR sérico (p = 0,561). Embora os pacientes com anti-DNA positivo tenham apresentado níveis mais baixos de suPAR urinário (p = 0,936), VEGF (p = 0,424) e suPAR sérico (p = 0,328) em comparação com aqueles com anti-DNA negativo, esse resultado não foi estatisticamente significante. As categorias de creatinina sérica e a relação proteína/creatinina não foram associadas aos níveis de suPAR e VEGF.

Confirmamos uma correlação positiva entre SLEDAI-2K e suPAR urinário (coeficiente de correlação de Spearman r = 0,347; p = 0,012, Figura 1), e também entre hematúria glomerular e suPAR urinário (r = 0,322; p = 0,021). Quanto maior os índices de SLEDAI-2K e hematúria, maiores os níveis de suPAR urinário.

**Figura 1.** Diagrama de dispersão bidimensional entre suPAR urinário (pg/mL) e escores SLEDAI-2K em pacientes com nefrite lúpica.

Não foi encontrada associação estatisticamente significante entre VEGF sérico e suPAR urinário (r = −0,197; p = 0,165), proteinúria (r = −0,187; p = 0,188), creatinina sérica (r = 0,034; p = 0,814) e relação proteína/creatinina urinária (r = −0,220; p = 0,121).

O mesmo ocorreu ao investigar possíveis associações entre o suPAR sérico e o SLEDAI-2K (r = 0,055; p = 0,699), os níveis de proteinúria de 24 horas (r = 0,154; p = 0,280) e a relação proteína/creatinina (r = 0,253; p = 0,073). Níveis mais elevados de creatinina sérica foram associados a níveis mais altos de suPAR sérico (r = 0,511; p < 0,001).

## Discussão

A nefrite lúpica é uma das manifestações mais graves do LES, resultando em alta morbidade e mortalidade. A avaliação da atividade e da gravidade da injúria renal é importante para um melhor acompanhamento dos pacientes. Este estudo teve como objetivo avaliar o perfil de suPAR e VEGF em pacientes com NL e sua eventual associação com a atividade da doença no LES e na própria NL.

A amostra da população com NL aqui avaliada consistiu em 51 pacientes, sendo 88,2% do sexo feminino, com idade média de 36,1 anos. Essas características são semelhantes às encontradas em outros estudos. Satirapoj et al.^13^ estudaram 68 pacientes asiáticos com predominância do sexo feminino (97,1%) e idade média de 31,7 anos. Em 2017, um estudo brasileiro analisou 8.761 causas de óbito nas quais o LES foi registrado como doença subjacente. Nesse estudo, 90,7% dos participantes corresponderam a mulheres, na faixa etária foi de 20 a 39 anos, e 49,2% eram brancos^14^.

A classificação racial/étnica dos indivíduos no Brasil é dificultada pela elevada miscigenação da população. No entanto, com base na raça atribuída aos pacientes nos prontuários médicos, 51% foram considerados brancos, uma porcentagem semelhante à do último estudo citado^14^.

Em nosso serviço, os pacientes com NL são geralmente submetidos à biópsia renal quando apresentam anormalidades urinárias mais proeminentes e/ou disfunção renal no momento da apresentação e/ou não respondem ao tratamento imunossupressor durante o acompanhamento. Com relação ao diagnóstico histológico, houve uma predominância de pacientes com NL classe IV (51%), seguidos por NL classe V (31,4%). A classe IV representa o padrão histológico associado às formas mais graves de NL, conforme relatado por outros autores^13, 15, 16^. Vale ressaltar que nosso estudo foi realizado em um hospital de cuidados terciários que recebe pacientes de todo o país, muitas vezes encaminhados por apresentarem condições graves e complexas, o que em parte justifica a predominância dessa apresentação. Embora a classe IV da NL tenha sido a classe histológica predominante, somente uma minoria desses pacientes (13,7%) apresentou IRA em algum momento do curso da doença.

Houve predominância de mulheres com NL classe IV e de atividade moderada na população do estudo (de acordo com o SLEDAI-2K). O SLEDAI-2K é uma modificação do SLEDAI original e ambos são usados como indicadores de mortalidade no LES, além de serem utilizados para mensurar a atividade da doença e avaliar a necessidade de início do tratamento^17^.

Neste estudo, observou-se uma correlação positiva e crescente entre os escores do SLEDAI-2K e os níveis de suPAR urinário (quanto maior o escore, maiores os valores do biomarcador urinário), mas o mesmo não ocorreu com os níveis séricos de suPAR e VEGF.

Em nossa população, o teste para anticorpos anti-DNA foi positivo em 33,3% e a hipocomplementemia (redução de C3 ou C4) ocorreu em 31,4% dos pacientes. Os níveis de complemento e a positividade para anti-DNA não apresentaram correlação com os níveis de suPAR ou VEGF. Sabe-se que títulos positivos ou crescentes de anticorpos anti-DNA e hipocomplementemia, especialmente níveis reduzidos de C3, são indicativos de atividade renal; porém, esses achados não são essenciais nem suficientes para tal diagnóstico^18^.

A hematúria glomerular isolada é uma anormalidade urinária importante no diagnóstico e acompanhamento de pacientes com NL e, neste estudo, a hematúria esteve presente em 54,9% dos pacientes no momento da coleta das amostras. Embora frequentemente associada à atividade renal, a hematúria nem sempre prediz doença ativa na NL^19^, e testes adicionais são necessários para estabelecer o acometimento renal decorrente da NL. A concomitância de suPAR urinário elevado e hematúria reforçaria a atividade renal secundária ao LES.

Na avaliação de 87 indivíduos com NL, foi encontrada uma correlação significante entre NL ativa e VEGF urinário, mas não com os níveis séricos de VEGF^6^.

Os biomarcadores aqui avaliados também foram estudados em outras glomerulopatias, como a glomeruloesclerose segmentar e focal. Li et al.^20^ avaliaram o suPAR sérico em 109 indivíduos e observaram que seus níveis foram significantemente mais elevados em pacientes com essa doença em relação a indivíduos saudáveis ou a pacientes com doença de lesões mínimas ou nefropatia membranosa.

No presente estudo, avaliamos biomarcadores laboratoriais tradicionais e novos em pacientes com NL. Não houve associação entre os níveis séricos de suPAR e VEGF e a atividade renal ou geral do LES, mas os níveis urinários de suPAR se correlacionaram com a presença de hematúria glomerular e com escores SLEDAI-2K mais elevados, ou seja, com a atividade da NL e do LES.

As limitações deste estudo incluem as peculiaridades da etnia brasileira que podem estar relacionadas a polimorfismos do gene VEGF e à suscetibilidade ao LES. Não foram avaliados subgrupos de pacientes de acordo com a duração da doença, tipos de tratamento, achados de biópsia renal e outros parâmetros, uma vez que seria necessário um número maior de pacientes para compor subgrupos representativos. Além disso, embora os pacientes tenham sido recrutados no início do curso da doença, com uma média de 2,5 anos desde o diagnóstico até a inclusão no estudo, diferentes períodos sob o uso de antimaláricos e/ou imunossupressores podem ter impactado os resultados.

Existem poucos estudos que investigam o papel do suPAR e do VEGF em doenças glomerulares, particularmente na NL. Esses resultados iniciais sugerem um potencial papel para o suPAR urinário como um biomarcador capaz de refletir a atividade da NL e do LES, que pode ser utilizado como uma ferramenta adicional na prática clínica, e é dosado em uma amostra de urina de fácil obtenção.

## Data Availability

Os dados que não foram apresentados aqui e que corroboram os resultados deste estudo estarão disponíveis mediante solicitação ao autor correspondente.
